# Dry Sanitation

**Published:** 1895-02-23

**Authors:** 


### DRY SANITATION.

Dr. Poore read a paper at the meeting of the Sanitary Institute, on February 13th, in which he advocated the dry system of sanitation as opposed to water-carriage. He pointed out that the conversion of the excreta into humus was mainly due to the action of mould fungi and aërobic micro-organisms, and that it was absolutely necessary that the material should be exposed freely to the air. For the proper growth of these microbes it was essential that there should not be present any excess of moisture, and therefore it became necessary to allow the urine to run away and be absorbed by earth or other absorbent material. When practicable, the best system was to bury the excreta daily in shallow furrows in a well-tilled garden. He spoke highly of the absorbent capacity of sawdust, especially that from deal, but peat or dry earth would answer as well. He said that the abandonment of the pail system by Nottingham and other towns was not due to its being sanitarily defective, but because it was cumbersome, expensive, and inelegant; and he maintained that the analysis published by the Rivers Pollution Commission with the view of showing that there was no material difference between the sewage of water-closet and midden towns could not be taken to support that view, for notwithstanding the enormous dilution of the water-closet sewage, it contained 20 per cent. more combined nitrogen and 23 per cent. more ammonia than the sewage of midden towns.

